# Can Citizen Science Assist in Determining Koala (*Phascolarctos cinereus*) Presence in a Declining Population?

**DOI:** 10.3390/ani6070042

**Published:** 2016-07-14

**Authors:** Emily Flower, Darryl Jones, Lilia Bernede

**Affiliations:** Environmental Futures Research Institute, Griffith University, Nathan 4111, Australia; d.jones@griffith.edu.au (D.J.); l.bernede@griffith.edu.au (L.B.)

**Keywords:** citizen science, citizen science guidelines, koala management

## Abstract

**Simple Summary:**

Current scientific methods used to determine national population estimates for species like the koala, where individuals are scattered over a vast area, have failed to deliver an accurate and widely accepted result. Current citizen science projects aimed at mapping koala sightings reported by the public all use different methods and store their data in their own databases, each collecting scattered pieces of a much larger puzzle. To bring these pieces together, this study developed guidelines for a national citizen science project highlighting the importance of using one single method for data collection, and in turn assisting in the development of a national koala population database.

**Abstract:**

The acceptance and application of citizen science has risen over the last 10 years, with this rise likely attributed to an increase in public awareness surrounding anthropogenic impacts affecting urban ecosystems. Citizen science projects have the potential to expand upon data collected by specialist researchers as they are able to gain access to previously unattainable information, consequently increasing the likelihood of an effective management program. The primary objective of this research was to develop guidelines for a successful regional-scale citizen science project following a critical analysis of 12 existing citizen science case studies. Secondly, the effectiveness of these guidelines was measured through the implementation of a citizen science project, Koala Quest, for the purpose of estimating the presence of koalas in a fragmented landscape. Consequently, this research aimed to determine whether citizen-collected data can augment traditional science research methods, by comparing and contrasting the abundance of koala sightings gathered by citizen scientists and professional researchers. Based upon the guidelines developed, Koala Quest methodologies were designed, the study conducted, and the efficacy of the project assessed. To combat the high variability of estimated koala populations due to differences in counting techniques, a national monitoring and evaluation program is required, in addition to a standardised method for conducting koala population estimates. Citizen science is a useful method for monitoring animals such as the koala, which are sparsely distributed throughout a vast geographical area, as the large numbers of volunteers recruited by a citizen science project are capable of monitoring a similarly broad spatial range.

## 1. Introduction

The popularity of citizen science has soared in the last 10 years, with non-government organisations (NGOs) and decision-makers utilising the geographic dispersion of a large volunteer base to enhance their ability to monitor and manage ecosystem services and “at risk” species [1,2,3]. This growth can largely be attributed to progressions in technology, primarily in data storage, website development, social networking sites, and smartphone applications [4], all of which provide citizens with an unprecedented access to science. The use of community volunteers in citizen science expands the geographical range covered, minimises the time spent collecting data, and reduces the cost of data collection [3]. Therefore, citizen science used in conjunction with traditional science research models provides a scale of data unattainable for traditional science methods alone.

This argument, that conservation management benefits from a combination of both citizen science and more traditional scientific approaches, is tested through the implementation of a case study aiming to determine an effective method for koala population estimation. A general impression of Australia’s national koala population is difficult to obtain as the local populations in the north and east of the koala’s range are rapidly declining, whereas those populations further south are currently managed for overabundance [5,6]. This presents a significant challenge for authorities attempting to develop a national koala management program. In addition, this problem is intensified by a lack of data, population monitoring and assessment methods, and koala population trends on a national scale [6]. By ascertaining an accurate population estimate, responsible agencies will have access to a superior data pool and can therefore tailor management plans for each region. To enable this, a national monitoring and evaluation program is required, in addition to a standardised method for koala population estimates [5,6]. The success of such a program requires the collection of sufficient data that would allow the long-term monitoring of population changes and their causes, and moreover an evaluation of the effectiveness of the corrective actions [6,7].

The specific objectives were to (1) develop guidelines for the success of a regional-scale citizen science project following a critical analysis of existing citizen science case studies; (2) measure the effectiveness of these guidelines by implementing a citizen science project, Koala Quest, with the objective of estimating the presence of koalas in a fragmented landscape; and (3) to determine whether citizen-collected data can augment traditional science research methods by comparing and contrasting the abundance and accuracy of koala sightings gathered by citizen scientists with data collected by professional researchers and government staffers.

### 1.1. The Evolving State of Citizen Science 

#### Citizen Science Typologies

Citizen science projects have traditionally been classified by the varying extent of public participation into “top-down” or “bottom-up” management approaches [1]. Top-down citizen science instructs volunteers in standard scientific data collection techniques, thereby ensuring that the information is useful for research projects and government monitoring [1]. These models are of use when looking to implement large-scale monitoring for the early detection of potential environmental issues, and are effective at generating long-term data sets which can then be assessed by scientific researchers [8,9,10]. Bottom-up models involve the public to a larger extent and actively include volunteers not only in data collection, but also in providing feedback on the results of the project they were involved in [4]. These collaborative models between the public and scientific experts are increasing in use and popularity among the scientific community, as they have shown to lead to more positive decision-making and policy change by responsible agencies and governing bodies than other management approaches [1,4,11]. The ability to retain participants will lead to an educated and experienced group of volunteers who may recruit citizen scientists themselves, further increasing the collection of quality data [4]. 

Traditional citizen science models require minimal assistance from the public as volunteers either present scientific researchers with a problem (scientific question), or act solely as the data collectors [4]. These models largely involve volunteers providing information to the central authority or researchers making decisions prior to informing the community of concern [1,4]. Citizen science projects may be categorised into typologies based upon the level of public participation employed (low, medium, or high), with traditional citizen science models involving low levels of public participation [4]. However, a shift has occurred in the operationalisation of citizen science from utilising low to high public participation management approaches, responding to the needs of the community and increasing communication between researchers and volunteers [1,12].

### 1.2. Benefits and Limitations of Citizen Science

The spatial range monitored during a citizen science project is much greater than what a small number of specialists could realistically achieve in the same timeframe, suggesting not only that citizen science projects will yield more data, but that they will also do so in less time [3,4]. Additionally, citizen science projects save funds by utilising community volunteers in place of professional scientists [13,14]. Furthermore, the use of citizen science as a research tool increases public awareness of environmental change and improves science literacy, while simultaneously fulfilling a broad range of scientific objectives [4,14].

A significant limitation facing many citizen science projects relates to the issues surrounding the quality of data submitted by volunteers, a point of contention among scientists, as some question the completeness and non-comparability of the data collected [15]. The potential for observer error and bias (and falsified reports) significantly increases when working with inexperienced volunteers [3,16,17]; however, a solution addressing most issues associated with data quality lies simply in the education of volunteers [18,19,20].

### 1.3. Koala Population Monitoring

#### 1.3.1. Background

Since European settlement, one of the greatest threats to Australian biodiversity has been the conversion of natural habitat into highly modified landscapes for either farming or residential purposes [21]. Soon after settlement, habitat loss and pelt hunting caused koala numbers to decline at an astonishing rate, ultimately altering their distribution [22,23].

Nationally, the International Union for the Conservation of Nature (IUCN) has classified koalas as of “Least Concern”; however, due to the rapid decline of numbers in southeast Queensland, koalas in this bioregion have been declared “Vulnerable to Extinction”. As of May 2015, this classification was extended to the entire state of Queensland due to the continued decline within local populations [24,25]. In contrast, koala management programs across the eastern states are currently challenged by overabundant populations in the southern area of the species’ range [5,6], presenting a unique conservation challenge in attempting to formulate a suitable koala management program and policy framework at a national level.

#### 1.3.2. Population Estimates

Koalas tend to have an unevenly clumped distribution, which, when combined with specialised habitat requirements, relatively low population numbers and their low detection rate, poses challenges when monitoring population and distribution trends [26,27,28]. The lack of koala population estimates at regional, state, and national levels reduces the ability to accurately monitor and assess management programs, which in turn hinders the goal of producing long-term management schemes for koala conservation [22,29]. To date, studies performed by government researchers and NGO koala organisations (e.g., [30,31]) have failed to provide comprehensive population data for an accurate and widely accepted national koala population estimate [29]. It is evident that professional researchers alone are neither able to collect sufficient data, nor do so in a timely manner. Numerous studies suggest that citizen science is able to overcome challenges associated with large-scale data collection by utilising volunteers from the public to report sightings over a vast spatial area in a greatly reduced timeframe, providing a dataset which may augment that gathered by professionals [3,4,13]. 

## 2. Materials and Methods

### 2.1. The Development of Guidelines for a Successful Citizen Science Project

Through a critical assessment of 12 citizen science papers (Table 1), specific guidelines for the implementation of a regional-scale citizen science project have been developed, with an interest in retaining participants for future studies. The papers reviewed were found using online databases such as the *Web of Science*, *GreenFILE*, *ScienceDirect*, and *ProQuest*, in addition to Google Scholar. Keywords used ranged from simple searches such as “citizen science” and “citizen science projects”, to more specific searches regarding the typologies of citizen science projects and their benefits and limitations. Papers were deemed suitable for review if they fell into one or more of seven categories: citizen science benefits, challenges, and recommendations; data validity; volunteer recruitment and retention; conservation methods and koala population distributions; types of citizen science; conceptual frameworks for future studies; and policy development.

### 2.2. Guidelines: Stages of a Citizen Science Project

Three distinct, progressive stages are required when implementing a citizen science project: (1) volunteer recruitment; (2) implementation of standardised methods; and (3) ensuring the quality and validity of the data is maintained.

#### 2.2.1. Volunteer Recruitment

The first stage of a citizen science study involves the project’s means of promotion, followed closely by the recruitment and training of volunteers. Traditional citizen science projects utilise radio, television, and newspaper advertisements as the primary method for promotion. When possible, recruiting public participants from an existing volunteer network will ensure an educated and experienced group of volunteers, serving to increase the number of submissions and the reliability of the dataset. Motivating and rewarding volunteers is key to retaining participants for future studies. 

Volunteers must familiarise themselves with the strict standardised sampling protocol to prevent the data from being rendered useless. To assist in the verification of data, volunteers are encouraged to provide a photograph of the target species when appropriate. If the target species of a project is difficult to identify, volunteers may be set an online test prior to their search to ensure they are capable of differentiating between any challenging aspects of identification. In the interest of volunteer retention, researchers should provide prompt feedback using language the participants will understand, and in a manner that ensures the anonymity of the volunteer is maintained.

#### 2.2.2. Data Validity

Ensuring volunteers receive sufficient training is crucial to the success of a citizen science project. Volunteers must be educated on the target species; including information on their appearance, habitat, diet, and the easiest method for in-field identification. It is vital to train all volunteers in the standardised sampling protocol to safeguard against unusable data. To ensure the use of the reported data, precautions must be taken to minimise the risk associated with public-gathered data. In addition, volunteers must be urged to choose search locations at random to prevent bias in data collection; volunteers may also be provided a range of example data collection sites representing all suitable habitat types. Importantly, participants must not report a false sighting if the target species was not sighted during their search, but instead report an “absence sighting”.

### 2.3. Koala Quest Project

Following the development of guidelines for a citizen science project, these stages were then trialled via the implementation of Koala Quest, a citizen science project designed to verify the efficacy of the guidelines. The model developed for this study involves citizens in the data collection process, a feedback report, and an emphasis placed upon the importance of volunteer retention for future studies of a similar nature; erring on the side of high public participation.

#### 2.3.1. Study Area

Data collection for Koala Quest occurred in southeast Queensland. The study area was originally intended to span the Moreton Bay, Ipswich, Brisbane, Redland and Logan local government areas (LGAs), but was later contracted solely to the Brisbane LGA due to a lack of sightings data from other regions. These shires comprise a fraction of the Koala Coast, a 375 km^2^ area in the southeast Queensland bioregion containing one of Australia’s most significant natural koala populations, due to its relatively large population located near a capital city, and its distinct genetic structure [30,32,33,34].

#### 2.3.2. Volunteer Recruitment

The project was promoted to the general public via the use of various media outlets, such as (1) flyer and poster installation; (2) communication networks of the project authors; and (3) online social media. Flyers were distributed around the Griffith University Nathan campus, in addition to areas of high foot traffic along bush walks in both north and south Brisbane.

#### 2.3.3. Data Collection

**Figure 1:**
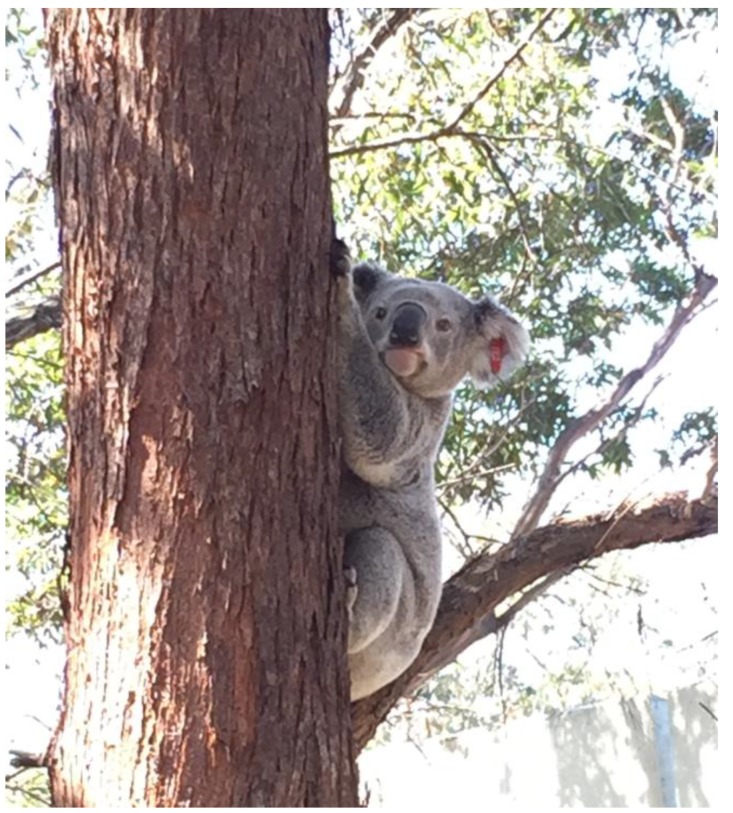
Koala sighting submitted by a volunteer.

Koala occurrence data were collected during the Koala Quest study held over two periods of four months (May–August) in 2013 and 2015, in addition to a single intensive week of koala spotting during the week of 6–12 July 2015. Volunteers were asked to report the (1) date the koala was seen (2) location of the sighting; (3) number of koalas observed; (4) health status of the koala (healthy, sick, injured or dead); and where possible; (5) a photograph of the sighting (Figure 1). Volunteers could report the sighting via one of four avenues; the Koala Quest 2015 Facebook page, email, BioTag smartphone application, or contact number. 

Volunteers were instructed to slowly walk a 100 m transect, pausing every 20 m to conduct a 360° search of all visible trees. Participants inspected the base and trunk of the trees for scats and scratches, before searching the branches and canopy for koalas. Volunteers were required to repeat this search for three transects, each spaced 100 m apart in order to avoid duplicate sightings.

#### 2.3.4. Data Validity

Citizen scientists were educated on the appearance of koala scratch marks on trees and also taught how to identify koala pellets in order to determine areas where koalas likely inhabit, while not physically observing a koala. Due to the koala’s status as a charismatic and well-loved national icon of Australia, minimal training was required for identification purposes. Data was cleaned and checked after each submission, with obvious duplicate sightings removed. Volunteers were also encouraged to report absence data (defined in this study as zero sightings during a sampling effort) to accurately determine the distribution of koalas in this area.

#### 2.3.5. Historical Data Sets

To effectively evaluate this study’s method for data collection, koala sightings were also gathered from Koala Tracker, a citizen science project, and the Department of Environment and Heritage Protection (DEHP), a government-led project employing a traditional research model. This allowed a direct comparison between citizen science and non-citizen science collected data, highlighting the most efficacious and efficient method for large-scale data collection when estimating an animal’s presence throughout a determined region. 

#### 2.3.6. Presentation of Results

To visually demonstrate the difference between sightings collected by citizen scientists and professional researchers over the past 10 years (2005–2015), maps were generated using ArcMap software (version 10.3, Esri, Redlands, CA, USA). These maps overlayed koala sighting coordinates with spatial data of southeast Queensland, such as road networks, proximity to green spaces, housing density, and koala habitat maps. The final analysis involved an assessment of the guidelines developed for a successful citizen science project at a regional scale.

### 2.4. Human Ethics Statement 

This research was approved by the Human Research Ethics Committee of Griffith University (Ref No: ENV/26/15/HREC).

## 3. Results—Locating Koalas in the Greater Brisbane Region

### 3.1. Comparison of Koala Sightings Collected from Three Data Sources 

**Figure 2:**
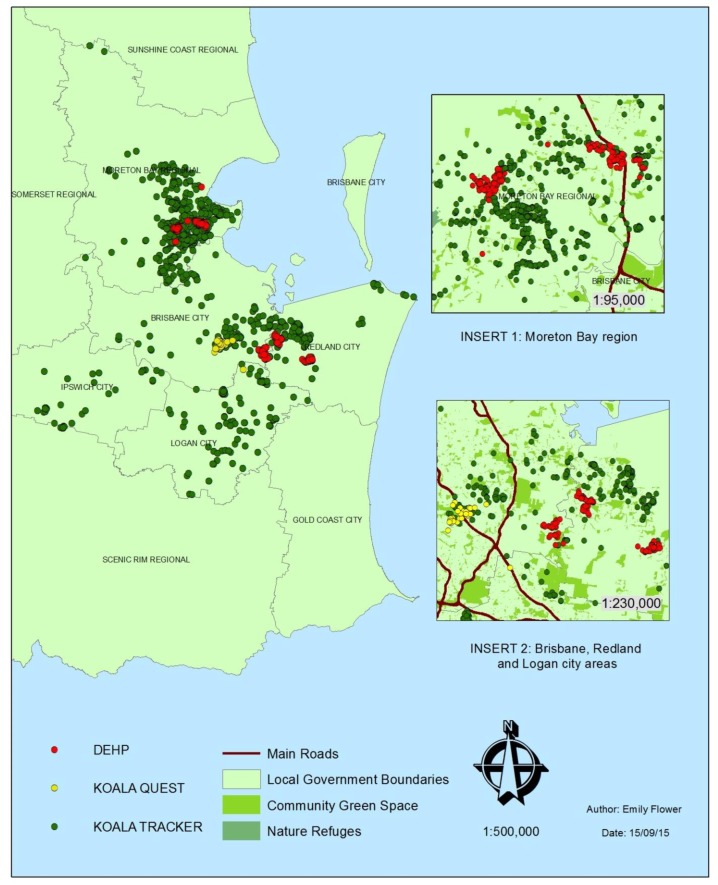
Data source comparison—Koala Quest, Department of Environment and Heritage Protection (DEHP), and Koala Tracker.

Figure 2 depicts a visual comparison of all data sources used in this study. Koala sightings were reported to Koala Quest over two four-month periods in 2013 and 2015, while citizens submitted sightings to Koala Tracker over a 10 year span, from 2005–2015. In addition, the data collected by DEHP ranges between 2005 and 2012. All 46 Koala Quest sightings (apart from one) were reported from within the Toohey Forest Conservation Park, located in Nathan, southern Brisbane. Social media collected 36 koala sightings, email yielded 10 sightings, and both the contact number and smartphone app did not record any koala sightings within the designated study area.

Sightings reported to the Koala Tracker website occurred throughout the Greater Brisbane region and were widespread throughout the Logan and Ipswich LGAs; however, clumping is evident in the Moreton Bay, Brisbane, and Redland LGAs. A significant portion of koalas in the Moreton Bay region appear to have been detected beside roads (Figure 2, Insert 1). In contrast, a large number of koalas in the Brisbane, Redland, and Logan areas were located in or near community green space or nature refuges (Figure 2, Insert 2). Koala sightings recorded by DEHP are aggregated into five distinct aggregations in the Moreton Bay, Brisbane, and Redland LGAs. Two separate clumps were evident in the Moreton Bay region, with one occurring in or near community green space, and the second found on either side of a main road (Figure 2, Insert 1).

**Figure 3:**
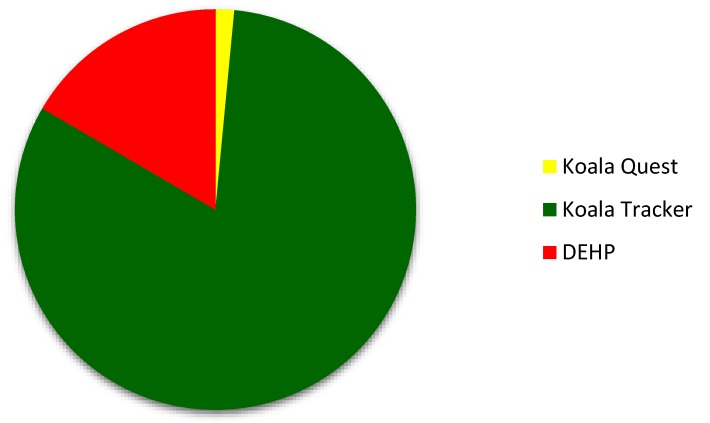
Proportion of sightings produced by each data source—Koala Quest, Koala Tracker, and DEHP.

The difference in the abundance of koala sightings between data sources is particularly evident in Figure 3, with Koala Quest, the smallest source, displaying 46 sightings, and Koala Tracker and DEHP reporting 2501 and 506 sightings, respectively. Citizen scientists reported almost 80% more koala sightings to Koala Tracker in 10 years (2501) than DEHP was able to collect in eight years (506) (Figure 3).

## 4. Discussion

### 4.1. The Process of Developing Guidelines for a Citizen Science Project

The guidelines developed were an amalgamation of widely accepted practices for a citizen science project and a number of more recently recognised elements, such as those associated with the evolving nature of technology. Citizen science projects are able to augment data collected by specialist researchers as they gain access to data which has previously been difficult or impossible to otherwise access [3]. Volunteers who remain with a study for a number of years allow researchers to identify trends in the data and to set conservation practices, as the quantity of data collected (within the comparatively marginal timeframe and extensive geographical region) would be unsustainable and unavailable due to the logistical constraints of the traditional scientific research model, without the use of citizen scientists [3,13,35]. The success of existing citizen science projects [1,2,4,36] suggests that these studies are capable of offering substantial and noteworthy contributions to the scientific community, and should consequently receive acknowledgement in the form of funding and the deserved attention from policy makers; however, a shortage of scholarly research currently remains despite an increasing number of citizen science projects applied across a wide range of fields [2].

#### 4.1.1. Volunteer Recruitment

The primary recruitment medium employed for Koala Quest was the social media website Facebook. Volunteers were encouraged and motivated to participate through the multitude of Facebook “posts” generated throughout the study, including koala “fun facts” intended to entice volunteer contribution. In addition to the use of social media platforms, Koala Quest was advertised through the installation of posters promoting the study throughout the Griffith University Nathan campus and walking tracks in areas of koala habitat.

The low numbers of citizen scientists participating in the Koala Quest project (*n* = 46) is most likely attributed to the ineffective recruitment capacity due primarily to a lack of resources; greater funding would enable better promotion and exposure to potential participants.

#### 4.1.2. Data Collection Protocol

Facebook posts detailing the standardised data collection procedure were posted online, describing the appropriate steps to be taken when searching for koalas. Despite these instructions, a large number of volunteers failed to correctly report a sighting to the various data collection platforms. This is likely due to the participant reporting a koala sighting in response to the poster advertisements, but overlooking the data collection procedure outlined on the Facebook page.

The BioTag smartphone app held high potential for the accurate record of a koala sighting as it prompted the user to enter all metadata prior to finalising the submission. Unfortunately, volunteers opted for alternative methods to submit reports, leading to an exchange of emails and messages in order to extract the required information.

Sightings reporting a scat or scratch mark were inexistent, in addition to any absence sightings. The lack of absence data may be attributed to the hesitation and bias attached to reporting naught study species sighted during a sampling effort [37].

#### 4.1.3. Data validity

Several issues were identified involving the validity and quality of the data collected by Koala Quest or the datasets sourced from Koala Tracker and DEHP. This is likely due to the small scale of the Koala Quest project, as the refined spatial scale and low participant numbers (*n* = 46) decreased the likelihood of experiencing data validity issues. Furthermore, the comprehensive reporting methods employed by Koala Tracker and the precision of the sampling procedures executed by DEHP staffers also contributed to quality datasets. As mentioned above, the main issues identified were the data related to the volunteers reporting a sighting without all necessary metadata present, and the complete lack of reported absence data. The training and education of volunteers not only improved data validity by reducing sampling bias when working with inexperienced public participants, but also educated the public about the scientific practices involved when undertaking a research project [35,38]. In addition, enforcement of the study protocol is imperative in order to ensure a quality dataset. This was achieved through the training of volunteers and the meticulous inspection of sighting submissions for erroneous errors and incomplete reports. Studies show methods such as a contribution leader board for volunteers submitting valuable data can be implemented, as this will consequently lead to an accurate and comprehensive management program [13].

### 4.2. Koala Quest, Koala Tracker and DEHP Data Source Comparison

A comparison may be drawn between the Koala Tracker and DEHP data sources regarding the quantity and reliability of the data collected over a similar time frame (eight to ten years) between specialist and non-specialist data collectors, and over the same geographical region. Therefore, they can provide an insight into whether citizen science is able to augment the scale of data collected by researchers and government personnel.

The difference between the number of sightings gathered by Koala Quest (46), DEHP (506) and Koala Tracker (2501) is apparent in Figure 2 and Figure 3, with citizen scientists using the Koala Tracker website reporting almost five times the koala sightings collected by DEHP government researchers. The stark contrast between the abundance of sightings collected by each data source emphasises the potential of a data collection program including both government researchers and citizen scientists. A national koala population estimate is likely to be achieved, providing a set of guidelines are developed highlighting the importance of standardised methods, as well as ensuring the volunteers are educated on the importance of data collection procedures [5,6].

### 4.3. Spatial Information Gained from Surveys

Figure 2 and Figure 3 present overwhelming support in favour of the argument that citizen science can add to, and enhance, data collected by researchers and government staffers [2,14]. The greatest factor restricting the effective management of the koala population as a whole is the lack of data collected, which consequently leads to the inability to provide nationally accepted population estimates. In 2004, Sullivan et al. collated estimates from the NGO Australian Koala Foundation (AKF) and various specialists, noting that the national koala population estimates ranged from 45,000–80,000 (AKF) to 255,000–310,000 [22,37,39]. The uncertainty surrounding a national koala population estimate continues to this day, as koala organisations and specialist researchers remain unable to agree upon a method of population estimation. Although some information is known about the populations of koalas found within the Koala Coast of east Australia [30], the abundance of koala populations in the southeast Queensland bioregion remains unclear, as complete datasets have not yet been created. A national population estimate is necessary in order to design an effective management program that not only deals with the declining numbers in northern New South Wales and Queensland, but also the overabundance issues in the southern areas of the koala’s range. This estimate would highlight the dire situation particular koala populations currently face, and would assist in mitigating exaggerated estimates. To collect the quantity of data required by specialists to effectively estimate koala populations, and to provide a sustainable method for research and monitoring programs, the use of citizen science may be a necessary addition to survey the remote areas and sparse distributions inhabited by koalas [3,13,35].

## 5. Recommendations for Future Studies

### 5.1. Volunteer Recruitment 

In future studies, a higher significance would be placed upon the recruitment methodologies employed to entice volunteers, such as the use of television, radio, and newspaper advertisements to ensure a large audience is reached [3,14]. It is critical to implement recruitment methods throughout the entire study area to engage the greatest number of potential volunteers, in order to yield a sufficient quantity of data, and to mitigate spatial sampling bias.

### 5.2. Data Collection Protocol

Koala Quest erred on the side of higher public participation due to the focus placed upon educating the citizen scientists on the plight of the koala in southeast Queensland, the provision of feedback to volunteers, the broad spatial scale, and the desire for the collection of valid scientific data. If this study were to be repeated, a greater significance would be placed upon the feedback provided to, and received from, the volunteers, increasing volunteer retention for future studies, and employing new methods for the intrinsic motivation of participants.

### 5.3. Data Validity

Quality data collection is also critical to the growth and validity of citizen science as it becomes a more widely accepted and valuable research tool [3,18]. In addition to standardised methods and the provision of feedback to volunteers, data quality concerns may be mitigated through the creation of a clear research question and data validity techniques, such as an automated electronic filter, minimising human error and increasing both the researchers’ and volunteers’ confidence in the quality of data produced [18].

A potential relationship between community-based monitoring efforts and a considerable improvement in ecological health has been highlighted, as projects involving higher volunteer participation appear to have a greater ability to positively influence biodiversity management [1,3,4]. This underlines the importance of community involvement when seeking to initiate environmental change, as the collaboration between citizens and researchers yields greater results and vastly improves the ability to monitor environmental concerns [4,10,40]. However, the success and efficiency of such programs relies upon the implementation and enforcement of standardised data collection methods and the education of volunteers.

## 6. Conclusions

This study demonstrates the significant contribution of citizen science, highlighting its potential to augment information collected by traditional scientific research models, and consequently provides an effective management scheme for “at risk” species. The role of citizen science has transitioned from merely a method for data collection, to models involving the adaptive management of urban environments [1,4]. These management schemes are reported to possess greater influence over conservation efforts and policy change as they are capable of offering substantial and noteworthy contributions to the scientific community, as demonstrated by a number of successful citizen science projects [1,2,4,36].

## Figures and Tables

**Table 1:** Studies reviewed for the development of guidelines for a small-scale citizen science project.

Author	Study Focus	Points Used towards the Development of Guidelines
**Bruce et al., 2014**	Educating volunteers in citizen science (CS) projects	- educate volunteers to reduce/eliminate sampling biases
**Bonter and Cooper, 2012**	How to ensure data validity (case study)	- how to minimise the risk of invalid data - automated filters
**Cohn, 2008**	Benefits of CS and gathered feedback from volunteers	- volunteer demographic and feedback - importance of training volunteers - study protocols must be enforced
**Conrad and Hilchey, 2010**	Literature review of past 10 years to find common benefits, challenges, and recommendations for successful CS projects	- types of CS and the benefits and limitations of each - management and monitoring types - provided a framework example
**Cooper et al., 2007**	Find a method to conserve wildlands in urban environments	- types of CS and the benefits and limitations of each - benefits and limitation of a traditional research method - provided a framework example
**Franzoni and Sauerman, 2014**	Systematic understanding of CS and conceptual framework and agenda for future research	- benefits and limitations of CS - volunteer recruitment and retention - volunteer feedback
**Hollow et al., 2015**	CS is useful for policy development and gathered feedback from volunteers (survey)	- advertisement information - specific information on a koala CS project - volunteer demographic
**Mulder et al., 2010**	Volunteer recruitment and retention strategies	- retain volunteers for future studies - recruit from an existing volunteer pool when possible - potential for human error - educate public
**Raddick et al., 2009**	Importance and benefits of CS—aims to make CS commonplace	- CS is a valuable research tool
**Sequeira et al., 2014**	Predicting species distribution (koala case study)	- advertisement information - benefits of CS - used their BioTag smartphone app for Koala Quest project - specific information on a koala CS project
**Silvertown, 2009**	Reasons for increased use of CS	- challenges and guidelines
**Wiggins and Crouston, 2011**	CS typologies–identified 5 types of project characteristics	- benefits and limitations of CS

Data collection protocol.

## Abbreviations

The following abbreviations are used in this manuscript:

DEHP: Department of Environment and Heritage Protection
IUCN: International Union for the Conservation of Nature
NGO: Non-Government Organisation
